# Collaborative governance of an integrated system for collecting contributions for social health insurance, pension, and taxes from the informal sector: a synthesis of stakeholder perspectives

**DOI:** 10.1186/s12913-024-11634-4

**Published:** 2024-10-17

**Authors:** Nelly Claire Muntalima, Adam Silumbwe, Joseph Mumba Zulu, Chris Mweemba, Peter Hangoma

**Affiliations:** 1 Department of Health Policy and Management, School of Public Health, University of Zambia, P.O Box 50110, Lusaka, Zambia; 2 Chr. Michelson Institute (CMI), Bergen, Norway; 3 Bergen Centre for Ethics and Priority Setting (BCEP), University of Bergen, Bergen, Norway; 4 Department of Epidemiology and Global Health, Umeå University, Umeå, Sweden

**Keywords:** Contribution collection, Integration, Informal economy, Social health insurance, Pensions, Taxes, Zambia

## Abstract

**Background:**

Many low-and middle-income countries have adopted social health insurance schemes. However, the collection of contributions from the large informal sector of these economies poses a significant challenge. Employing an integrated system of contribution collection from all relevant institutions may be cost-effective. We used the integrative framework for collaborative governance, to explore and explain factors that may shape the governance of an integrated system for collecting contributions for social health insurance, pension, and taxes from the informal sector in Zambia.

**Methods:**

We undertook a qualitative case study involving 25 key informants drawn from government ministries and institutions, cooperating partners, non-governmental organizations, and association representatives in the informal sector. Data were analyzed thematically using Emerson’s integrative framework for collaborative governance.

**Results:**

The main drivers of collaboration included a need for comprehensive policies and legislation to oversee the integrated system for contribution collection, prevent redundancy, reduce costs, and enhance organizational effectiveness. However, challenges such as leadership issues and coordination complexities were noted. Factors affecting principled engagement within the collaborative regime consisted of communication gaps, organizational structure disparities, and the adoption of appropriate strategies to engage the informal sector. Additionally, factors influencing shared motivation involved concerns about power dynamics, self-interests, trust issues, corruption, and a lack of common understanding of the informal sector.

**Conclusion:**

This study sheds light on a multitude of factors that may shape collaborative governance of an integrated system for contribution collection for social health insurance, pension, and taxes from the informal sector, providing valuable insights for policymakers and implementers alike. Expanding social health insurance coverage to the large but often excluded informal sector will require leveraging factors identified in this study to enhance collaboration with pension and tax subsystems.

**Supplementary Information:**

The online version contains supplementary material available at 10.1186/s12913-024-11634-4.

## Background

Governments in Low- and middle-income countries (LMICs), face substantial healthcare financing challenges hampering attainment of universal health coverage (UHC). This was further worsened by the flattening donor resources during the COVID-19 pandemic, posing a significant obstacle to delivering quality healthcare [1]. To achieve UHC, LMICs are moving away from healthcare financing models that depend on out-out-pocket payments to pooling based systems where everyone contributes to healthcare irrespective of whether they fall sick or not [2]. Pooling-based financing systems such as the Social Health Insurance (SHI) enhance population access to quality healthcare with minimal financial hardship [3]. However, many sub-Saharan Africa (SSA) countries fail to pool sufficient revenue owing to poor compliance and inefficiency [4], worsened by a large share of the informal sector [5]. This sector comprises entities that operate outside government laws and regulations, characterized by casual labor, self-employment, unregistered businesses, and a lack of formal structures, making them difficult to tax [6]. Covering the informal sector using the prepayment systems is challenging because of their limited capacity to pay [7]. In addition, this group is difficult to identify and engage, while the lack of social security and the irregular nature of employment makes it difficult to assess their income [8, 9] .

Collecting contributions from the informal sector is not only a challenge for health but also for the broader tax systems, including income tax and contributions to other social protection schemes such as disability insurance and pensions [5]. For any state entity, the administrative and institutional capacity required to collect contributions or taxes from the informal sector may be too costly or even unattainable given their characteristics [9]. It has been argued that a collaborative system, where several institutions work together and create an integrated system for collecting contributions and taxes from the informal sector has the potential to be more cost-effective [10–12]. An integrated system enhances efficiency and compliance, promotes equity, and improves overall contribution mobilization by leveraging resources across different subsystems [13]. In this study, we view integration as “the linking or sharing of information, resources, activities, and capabilities by organizations in two or more sectors to achieve jointly an outcome that could not be achieved by organizations in one sector separately [14]”. A strategy used to bring about this collaboration, or more generally integrate the goals, interests, and actions of multiple stakeholders is termed collaborative governance [15].

Like other LMICs, in an effort to attain UHC, Zambia adopted the national health insurance scheme through National Health Insurance Act No. 2 of 2018 [16]. This Act prescribes that 1% contribution should be collected every month from all those in formal employment. However, more than 87% of the labor force is employed in the informal sector as domestic workers, bus and taxi drivers, and marketeers, traders, as well as micro and small enterprises owners. The overall insurance penetration rate is a meagre 3% of the population [17], while the pension contribution compliance rate of the informal sector is less than 5%, and the tax compliance among formal medium small and micro enterprises is only 30% [18]. Meanwhile, the National Pensions Scheme Authority, an entity responsible for collecting pension contributions, with support from the International Labor Organization, has been implementing measures to incorporate informal sector employees in the social security system, with minimal success [19]. Similarly, the Zambia Revenue Authority, a body that collect taxes has been unable to capture the informal sector for several decades [18].

Our study explores stakeholder perspectives on the feasibility of an integrated system for collecting taxes, contributions for SHI and pensions from the informal sector. We also explore possible collaborations between the state sectors and informal sector actors—such a traders’ associations—who could be instrumental in facilitating contribution collection and specific collaborative actions. Notably, studies on SHI and the informal sector have focused on establishing the willingness to pay [20–24], or uptake [25]. Others have assessed which prepayment scheme informal sector workers would prefer [26], or factors affecting demand for SHI [25, 27–30]. These studies generally find low uptake or demand for SHI, and this could be related to a lack of trust in governance systems. One of the studies showed that informal sector workers had serious concerns about the high levels of corruption [26]. Additionally, several studies have discussed how to extend tax collection or pension coverage to the informal sector [31–33]. However, most studies do not look at the feasibility of an integrated system for collecting tax, pension and SHI from the informal sector, particularly in a LMIC such as Zambia, with a large informal sector.

To address this gap in research, we applied an integrative framework for collaborative governance, to explore and explain factors that may shape governance of an integrated system for collecting contributions for SHI, pension, and taxes from the informal sector in Zambia. This study may provide valuable insights to other LMICs struggling to collect SHI contributions from the informal sector in a cost-effective manner.

### Theoretical framework

**Fig. 1:**
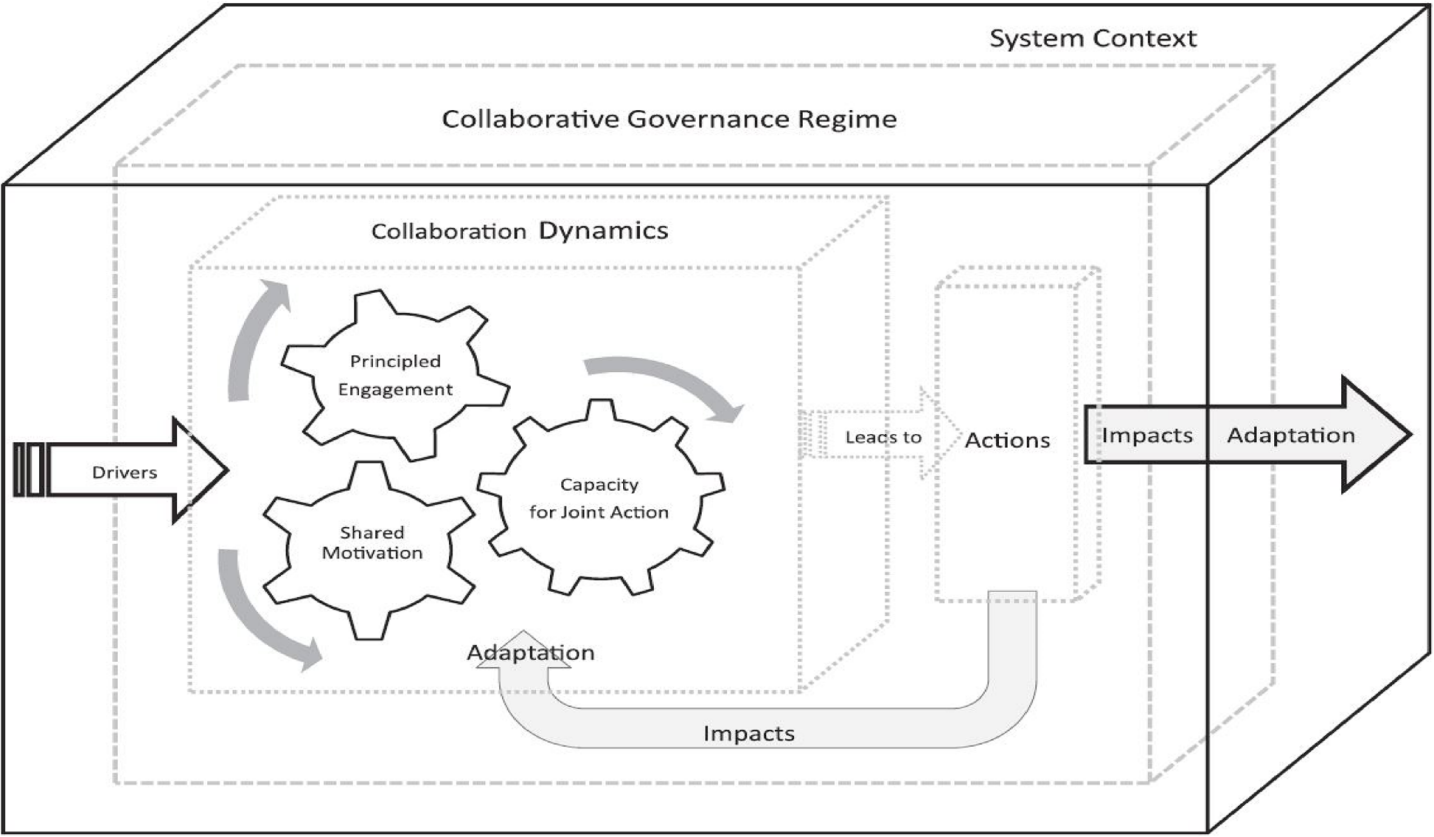
Integrative framework for collaborative governance [34]

Collaborative governance is a process of public policy decision making and management that engages across sectors to carry out a public purpose [14, 15]. We adopted Emerson’s integrative framework for collaborative governance to unpack potential factors that may shape governance of integrated system for collecting contributions for social health insurance, pension, and taxes from the informal sector (Fig. 1) [34]. This framework consists of three nested dimensions – the *general system context –* political, legal, socioeconomic environment, the *collaborative governance regime (CGR) –* a central feature of this framework – and its *collaborative dynamics and actions*. The system context creates opportunities and limitations and affects the dynamics of collaboration at the beginning and over time. Based on the system context, the *drivers* such as leadership initiate collaboration or the CGR. Collaborative dynamics comprise the interactive components of principled engagement, shared motivations and capacity for joint action that in the long run shape the effectiveness of the CGR. Our CGR of interest is the integrated system for collecting contributions in Zambia. Specifically, we focus on three components of the framework – the drivers of collaboration, and specific collaborative dynamics of principled engagement and shared motivation within this CGR. *Principled engagement* is crucial to setting in motion the rolling wheels of collaboration while *shared motivation* is the social capital holding the CGR together consisting of concepts such as mutual trust, mutual understanding, and commitment. Exploring these dimensions of the framework illuminates potential challenges to collaboration within and outside the integrated system and explores ways of addressing them.

## Methods

### Study design

We adopted an exploratory qualitative case study [35]. This design was chosen to gain in-depth insights into a relatively unexplored and complex phenomenon of integrated contribution collection for SHI, pension, and taxes from the informal sector in Zambia. The case study comprised key state and non-state actors who have or may have a role in the governance of this integrated system.

### Study setting

We conducted the study in Lusaka, the capital city of Zambia, where most key state and non-state actors responsible for the management of tax, pensions, social insurance, and formalized informal sector groupings are based. These actors included the Zambia Revenue Authority, the National Pension Scheme Authority, the National Health Insurance Management Authority, and informal sector organizations such as the Marketers Association of Zambia. Additionally, due to Lusaka’s position as the largest economy in the country, many people migrate there, seeking limited employment opportunities and often end up engaging in small-scale informal businesses.

### Participant selection and recruitment

We applied both purposive and snowball sampling methods. Recruitment began with respective government ministries overseeing taxation, pension, and health insurance in Zambia (Table 1). We sent interview requests to the Ministry Permanent Secretaries from Finance, Health, Labour and Social Security, Local Government and Community Development, who assigned appropriate individuals for interviews. After interviewing these state actors, we used snowball sampling to include quasi-government institutions, non-governmental organizations (NGOs), cooperating partners, and informal sector associations based on recommendations from the initial state actors. Initially, we purposefully selected 15 key informants with relevant knowledge of Zambia’s social security systems and potential integration structures for collecting contributions from the informal economy. We reached saturation, where no new information emerged, with 20 key informants, but added five more to ensure all relevant information was captured.

**Table 1:** Key informant categories and organizations

Category of Stakeholder	Organization	Participants Interviewed
Cooperating partners	World Bank, International labor organization	2
Government ministries	Ministry of health, Ministry of Labor, Ministry of Local Government, Ministry of Community development and Social Welfare	8
Non-governmental organizations	Financial Sector Deepening Zambia, Policy Monitoring and Research Centre, Systems for Better Health, Clinton Health, Access Initiative	3
Informal economy associations	Association for Employers of Domestic Workers, Domestic Workers Union of Zambia, Bus, and Taxi Drivers Association of Zambia, Marketeers Association of Zambia	7
Quasi-government institutions	NAPSA, ZRA, Zambia national farmers union, Zambia National Public Health Institute	5

### Data collection

We collected data using key informant interviews, employing three separate guides tailored for government officials, officials from cooperating partners and NGOs, and officials in the informal sector (Supplementary file). The guides were developed by the study team based on extensive literature review and prior research experience applying the integrative framework in Zambia [36, 37], and included questions on the drivers of collaboration, and collaborative dynamics which covered engagement modalities with the informal sector and factors shaping shared motivation, such as mutual trust and legitimacy. Each interview lasted about 45 min to an hour. We audio-recorded all interviews, taking notes only when respondents declined to be recorded. The collected data was transcribed verbatim in preparation for analysis.

### Data analysis

We used thematic analysis in NVivo 12 Pro Software, which is an approach that groups portions of texts in the transcripts into notable and related patterns [38]. The analysis was deductive in that the main themes – drivers of collaboration and collaborative dynamics of principled engagement and shared motivation were drawn from the study framework. It was also inductive in that we read the transcripts and the field notes in detail to identify sub-themes that were later grouped in appropriate main themes. This process culminated into the development of the code-list that was initially informed by preliminary reading of the first 6 transcripts by the first and second author and iteratively discussing the grouping of sub-themes with the rest of the research team. This coding structure was then imported to NVivo for coding of the rest of the transcripts (Table 2).

**Table 2:** Main themes and sub-themes

Main themes	Sub-themes
1. Drivers of collaboration	▪ A need for comprehensive policies and legislation ▪ Avoid duplication and cost reduction ▪ Improved organizational effectiveness ▪ Expanded coverage of the informal sector ▪ Leadership and coordination challenges
2. Factors affecting principled engagement	▪ Lack proper communication channels ▪ Differences in organization structures ▪ Inappropriate strategies for engaging the informal sector
3. Factors influencing shared motivation	▪ Fear of losing power and self-interest ▪ Lack of trust and corruption ▪ Lack of common understanding of what and who constitutes informal sector

### Ethical considerations

The study received ethical approval from the Biomedical Research Ethics Committee of the authors’ institution (REF. No. 035-06-17). Signed informed consent was obtained from all participants before each interview. Permission to record the interviews was also requested and the reason for recording was clarified. Before commencing the interviews, participants were reminded that participation was completely voluntary. To protect confidentiality and ensure privacy, participant codes to label the data were used instead of actual names. All methods were carried out in accordance with relevant guidelines and regulations.

## Results

The results are structured around three main themes from the study framework – drivers of collaboration, collaborative dynamics of principled engagement and shared motivation. Firstly, under drivers, sub-themes included the need for comprehensive policies and legislation, avoid duplication and reduce costs, improve organizational effectiveness, expand coverage to the informal sector, and leadership and coordination challenges. Secondly, within principled engagement, sub-themes encompassed lack of proper communication channels, organizational structure differences, and a need to adopt appropriate strategies to engage the informal sector. Lastly, shared motivation sub-themes included fear of losing power, lack of trust and corruption, informal sector willingness to contribute and lack of common understanding on what and who constitutes the informal sector.

### Drivers of collaboration

#### A need for comprehensive policies and legislation

The stakeholders from the government and non-governmental sectors emphasized the insufficiency of the current legal and policy framework to foster collaboration among them. Firstly, they contended that the existing policies and laws do not mandate the informal sector to participate in any social security schemes, with contributions predominantly relying on voluntary compliance. A representative from the non-governmental sector explained.

“…it would be helpful to put in place legislation that would compel institutions to put their information together…there is nothing that compels even ministries to work with other ministries. There is nothing that compels institutions dealing with social security to work with the revenue administration. So even before we talk about an integrated systems approach, we first need to consider the legislation that is there…otherwise we will come up with brilliant frameworks, but they wouldn’t work….” (P23, non-governmental organization).

Secondly, they lamented the absence of policies and laws mandating various institutions to exchange data pertaining to the informal sector. They asserted that data collaboration legislation was pivotal to effectively govern an integrated system for contribution collection. Further, enacting suitable legislation would not only encourage collaboration among institutions, but also guarantee the ethical and responsible management of the informal sector data as another government official described.

“…one of the issues with regards to data policies is that we currently do not have any data policy that would allow people or that would compel people or organizations to put their records together. It could be because of several reasons. I mean several organizations would have their reasons for instance the financial intuitions would have their reservations having to share information…” (P7, government ministry).

#### Avoid duplication and cost reduction

According to the stakeholders, there was more value in having an integrated system of contribution collection than the current silo approach. They reported that having an integrated system is beneficial as it would enhance performance by reducing duplications and administrative costs as well as strengthening partnerships. One quasi-government institution official had this to say.

‘…Collaborating among different organizations or ministries for contribution collection … would avoid duplication of work and…as a result more funds would go into ensuring the system works and less funding for management and administration…’ (P18, quasi-governmental organization).

Another representative from a government ministry added.

…I think that collaboration is the way to achieve the desired outcome for all organizations that would want to work together to capture the informal economy of Zambia. It would be a ‘win-win’ situation which is very attractive currently. You need to leverage on partnerships because going into it alone would involve the cost element, reinventing the wheel where it’s not necessary, duplicating efforts, etc.…” (P5, government ministry).

#### Improved organizational effectiveness

Majority of the stakeholders felt that different state institutions have specialized skills to bring to the table. For example, some state institutions were already reaching the informal sector through banking, telecommunication, pensions, tax and other services. Formal collaboration would not only enable each institution to allocate resources to activities in which it excels, but also improve coordination and effectiveness within the integrated system. A representative from a quasi-governmental institution commented on this perspective.

“…different organizations have their strengths… I think we have in most cases very intelligent people working in some of these organizations and so collaborating to create one system would really be beneficial. I would like to think that we would see a case where talent, energy and commitment are put together for a common purpose …” (P13, quasi-governmental organization).

#### Expanded coverage of the informal sector

The stakeholders recognized that an integrated system would facilitate knowledge sharing and dissemination, leading to improved decision-making and the ability to reach a larger portion of the informal sector. However, they voiced concern about the potential reluctance of certain organizations to participate in knowledge sharing initiatives.

‘…several ministries and institutions appear to be working in silos with different objectives and own targets to meet… so even when it comes to creating an integrated system, information that would be needed is seated in different ministries. For the informal sector you would need the Ministry of Finance to compel NAPSA, ZRA to provide information even for Ministries like agriculture to access that information…you need the Ministry of Health to come on board, ministry of information, transport and communication to come in but if they have their own objectives then integration becomes problematic…’ (P24, Non-governmental Organization).

#### Leadership and coordination challenges

Lack of leadership and coordination challenges, both at the broader system context and within organizations, was cited as a threat to collaborative governance. Stakeholders felt that collaboration requires strong leadership and coordination platforms. They indicated that past efforts to work together have proven futile due to coordination failure as pointed out below.

‘…we have had cases in the past where working groups were created to try and work on issues that were cutting across different sectors. It’s always the case that in the first days that people would attend and then later just stop showing up all together. This was even the case when there was a discussion on the social security policy a few years ago and the national health insurance is being a part of it. ……. people have pulled out and want to do things on their own…’ (P17, Government Organization).

### Factors shaping principled engagement

#### Lack of proper communication channels

Many stakeholders emphasized the need for respectful and open discussions when considering the establishment of an integrated contribution collection system. They asserted that transparent and honest communication should be maintained across all organizational levels right from the outset. Additionally, some underscored the significance of maintaining consistent and ongoing communication as a crucial element in any collaborative effort. A non-governmental organization stakeholder narrated,

‘…without consistent communication, using an integrated system would be challenging. The stakeholders that could collaborate would need to have a working group of some sort. This…well…could involve in-person monthly meetings, and some form of online platform on which they can share information just, so they keep each other in the loop…’ (P10, non-governmental organization).

#### Differences in organizational structures

Stakeholders held that differences in the organizational structures are a potential hindrance to collaborative governance. This is because ministries and quasi-government institutions who may have to collaborate have different regulations, norms, and structures for decision making. For example, decision on pensions may just involve the Pensions Authority, a quasi-government institution, while decision on taxes would first begin with the Revenue Authority and then move to the Ministry of Finance. They indicated that such differences may lead to frustration among collaborating institutions and hinder stakeholder engagement. This could also generate conflict, tension and may compromise shared commitment to the idea of collaboration.

‘…institutional differences among the collaborating parties…could be a possible source of conflict or tension as each actor has its own way of doing business…some risks need to be considered during the collaboration.’ (P8, Non-governmental Organization).

#### Appropriate strategies for engagement with the informal sector

Stakeholders representing informal sector associations favored the concept of integration. They pointed out that consolidating payments into a single transaction would be more convenient for them, as opposed to dealing with various organizations collecting contributions at different times.

” …honestly, our people would be more agreeable to making a once off payment as compared to having different organizations approaching them to collect contributions. Different organizations approaching them for contributions or taxes would cause distrust and resentment. In the end people will do whatever that can to avoid contributing…” (P20, Informal sector association).

The stakeholders believed that for an intergrated system for contribution collection to be efficient, the informal sector should be enganged at two distinct levels. Firstly, at the grassroots level, targeting small geographical units, and engaging with community structures. Secondly, at a broader level, targeting occupational groupings or associations. In the case of community structures, individuals would make contributions either within their community, facilitated by figures like a headman, or through a local health facility.

‘…community-based solidarity models seem to have achieved more compared to any other model…houses are clustered together, and these households contribute towards a local health facility. This brings about the issue of accountability…’ (P10, non-government Organization).

In addition to community structures, stakeholders proposed the utilization of informal sector associations. In this scenario, collection would be organized into smaller pools based on sectors. For example, farmers would have their designated pool, bus drivers, domestic workers and marketeers would have theirs.

“…We have to use the associations based on activity level. For example, you bring the marketeers together or carpenters but of course we need to ask what incentives they will have to be given…” (P6, government ministry).

Apart from informal sector associations, stakeholders stated that other quasi government and government institution already have systems that could be used to register the informal sector. For example, bus and taxi drivers could be captured under the Road Transport and Safety Agency which collects biodata as people obtain driving licenses:

“…leveraging partnerships is one of our key strategic elements. We have identified the key stakeholders. So, like for the bus and taxi drivers we do realize that institutions like RTSA is one of the key stakeholders and within that are also sector associations that represent the interest of the bus drivers…” (P18, quasi-government institution).

Farmers make up the greatest share of the informal sector. Apart from using associations and community/village structures, stakeholders highlighted the potential use of the e-voucher system to capture farmers, as one participant pointed out.

“…more than one million farmers are registered under the Farmer input support program and benefit from the e-voucher platform. This is a platform that can easily be utilized to capture the people of interest…” (P19, informal sector associations).

### Factors influencing shared motivation

#### Self-interests and fear of losing power

The stakeholders observed that certain individuals benefit from the existing status quo, where there are incentives for maintaining separate models of contribution collection. They stressed that the reluctance to commit to an integrated systems for contribution collection by some institutions is, in part, driven by self-interest. A government Ministry official recounted.

‘…you know we have to bear in mind that some people or institutions would naturally be against National Health Insurance because they would want to protect their interests. Because of this, we wouldn’t expect to see any commitment from them.’ (P19, Government Ministry).

Importantly, some stakeholders noted that organizations might be hesitant to collaborate due to the fear of relinquishing their power. A representative from a partner organization echoed this sentiment.

‘…quite frankly the challenge we have is that people will not want to work together even though this is what will be beneficial in the long run. This is due to mainly people thinking they will lose their positions or their jobs when one system is developed and used. It is merely for personal reasons because they think they will no longer be Directors or CEOs and that they will now have to start reporting to someone else.’ (P1, Cooperating Partner).

#### Lack of trust and corruption

Stakeholders exhibited a lack of trust in some institutions that may need to collaborate, attributing this skepticism to high levels of corruption. They expressed apprehension that an integrated system of contribution collection could lead to one organization misusing funds intended for other collaborating sectors. A cooperating partner stakeholder shared the following perspective.

‘…. like let’s say someone is paying the premiums for the fund at the University Teaching Hospital (UTH), the managing director for UTH can just go to the fund and say how much did you make today, give me some. So, we need to think of ways in which checks, and balances can be put in place to avoid such things.’ (P3, Cooperating Partner).

The lack of trust and apprehension about corruption is rooted in historical experiences, as recounted by a representative from one of the partner organizations.

‘…there have to been too many scandals…for example the scandal of 2009 and just recently with the Global fund money. There have also been very disturbing reports from the Auditor general. The money that is normally allocated from the national budget could go further if the systems were working better…’ (P4 Cooperating Partner).

Moreover, the stakeholders emphasized the necessity of instilling confidence in the newly established national health insurance scheme for it to be effective. Ensuring that people received services commensurate with what they contributed, was deemed crucial in addressing the lack of trust as shared by a non-governmental representative.

“‘…I think one of the controversies of the National Health Insurance is that you will start collecting from people for something that is not available yet because there is no money necessarily yet to increase what is already available at the facilities for them. They want to use the…. people will be paying but will not be getting anything and so many people are still in conflict with that…’ (P15, non-governmental organization).

#### Lack of common understanding of what and who constitutes the informal sector

Stakeholders indicated that different organization don’t share a common understanding of who constitutes the informal sector and their capacity. There was a belief by some organizations that the informal sector constitutes poor people and any effort to collect contributions from this sector may not bring economic value.

‘…every time we have brought up the idea of going into the informal sector even when it has to do with issues with collecting revenues, people automatically assume we are talking about the poor people. But we have people in the informal sector for example car dealers who make a good K20, 000 and just go under the radar… there is quite a significant of revenue lost…’ (P10, Non-governmental Organization).

## Discussion

This study is the first, to the best of our knowledge, to shed light on the factors that could shape governance of integrated system for collecting taxes, pension, and insurance from the informal sector. Specifically, our paper makes two significant contributions to the literature. First, using data from Zambia, we explore whether theoretical insights on collaborative governance from developed countries extend to developing contexts. The literature on collaborative governance attempts to explain how collaborative relationships function and what factors influence their success [15, 34]. However, since developing countries such Zambia often have weaker democratic consolidation and oversight institutions, it is unclear if these theoretical insights apply [39]. Second, we provide evidence of potential obstacles and necessary strategies to integrate the informal sector. In the following, we discuss our findings in relation to drivers of collaboration, and the collaborative dynamics of principled engagement and shared motivation, respectively.

Most of the stakeholders agreed that collaborating to implement a unified contribution collection system would be a cost-effective way of capturing the informal sector. This would minimize duplications, save scarce resources and ultimately enhance service delivery to the informal sector. Consistent with these views, studies have shown that collaboration could result in better utilization and pooling of resources [40, 41]. Other studies have found that an integrated system for contribution collection can increase compliance and coverage of the informal sector because of the streamlined processes [42, 43]. Yet there is evidence that there are problems in collaboration between state, non-state actors and the informal sector responsible for governing such systems in most African countries [44]. Our findings suggest that ethical and accountable leadership could enhance collaboration among key actors in an integrated system for contribution collection. Leadership is crucial to driving and maintaining collaboration, and is required at system level, cross-organizational level (inter), and within the integrated system (intra) as highlighted in the study framework [34]. In Uganda for instance, the role of political, inter and intra sector leadership is underscored in the successful governance of the multisectoral AIDS response in the National AIDS Council [45].

The stakeholders emphasized the importance of developing specific legislation that requires state organs to collaborate and establish minimum structures for effective communication. These structures could for example include coordination mechanisms and data-sharing platforms across SHI, pensions and tax sub-systems. In Portugal, legislation was enacted to initiate collaboration among ministries for implementation of healthy diet strategy [46]. However, ensuring the implementation of this intervention and coordinating collaborative efforts required additional steps. A Technical Working Group (TWG), comprising representatives from different ministries, was formed to oversee implementation. This TWG facilitated the implementation of various collaborative actions, such as imposing taxes on sugar-sweetened beverages. Conversely, our findings suggest that a structure such as TWG may not be as effective in coordinating contribution collection for Zambia due to a history of limited success, contributing to stakeholder skepticism about its effectiveness. In addition, TWGs tend to be weaker and work better as policy advisory bodies. Coordination structures, whether TWGs or others, must be established where they are most effective. In Kenya, it was found that establishing an inter-ministerial collaborative structure within a ministry posed challenges like inability to fund collaborative activities [47]. Cross-cutting collaborative and coordination structures must provide all actors within the integrated system a sense of ownership and belonging, to increase the likelihood of success.

Differences in organizational culture are pivotal in determining collaboration within integrated systems for contribution collection from the informal sector. Wiart et al., identified significant disparities in organizational culture, structures, and processes in Canada, underscoring the need for policymakers to comprehend these differences to effectively enhance collaboration [48]. Obstacles such as lack of trust, fear of losing power, self-interests, and inimical organizational cultures must be addressed to achieve effective governance. Integrating the informal sector faces a notable barrier in the form of the low trust in government, hindering the successful implementation of contribution collection systems. In Kenya, Okungu et al., highlighted that informal sector workers’ low confidence in governance mechanisms were due to concerns about corruption and the absence of punishment to perpetrators [26]. The stakeholders in our study proposed adopting information and communication technology-based system to mitigate corruption concerns. This underscores the potential role of technology-focused collaborative actions within an integrated system for contribution collection. In Kenya, the National Hospital Insurance Fund witnessed a 500% increase in voluntary payment subscribers between 2009 and 2017 after enabling payments via M-PESA, a mobile money platform [49].

Our study reveals that group associations and community structures could serve as effective channels for reaching the informal sector. By enabling contributions through associations within their occupations or at the community level, individuals could participate in an integrated system more readily. However, for this approach to succeed, collaborating institutions must be held accountable and demonstrate credibility by delivering anticipated benefits. A survey of 800 informal workers in Zambia found that the likelihood of tax compliance increases when the benefits of those taxes are directly experienced [50]. Health insurance benefits, for example, are more tangible and hence more likely to be accepted among informal sector workers compared to general taxes. Bundling health insurance with taxes could potentially enhance tax collection in the informal sector while reducing administrative costs for the SHI system and all involved organizations. However, effective stakeholder consultation and engagement would be essential, as highlighted in the literature [36], to foster trust and establish authentic partnerships [51].

### Strengths and limitations

This study boasts several strengths. It stands out for its comprehensive data collection involving active stakeholders deeply involved in working with the informal sector – both from government agencies, quasi-governmental and informal sector associations. This not only adds richness to the perspectives explored but also facilitates the triangulation of views from diverse sector representatives. The data analysis process, characterized by reflection and iteration, enhances the validity and trustworthiness of our findings. The codes and themes were not only identified, but also subject to collective discussion, modification, and agreement by the entire research team, ensuring alignment with the study question. Further, the incorporation of an integrative framework introduces a perceptive dimension to the examination of collaborative governance of an integrated system for collecting contributions for social health insurance, pension, and taxes from the informal sector. However, it is essential to acknowledge certain limitations. This study is constrained by its focus on specific stakeholder groups, namely social security providers, NGOs, cooperating partners, associations of informal sector workers, and tax administrators. Notably, ordinary workers in the informal economy were not directly included in the study. While their representatives, who are informal sector workers themselves, were incorporated, there remains a possibility that the perspectives of ordinary workers may differ and could have added valuable insights not captured in the current study.

## Conclusion

Our study explored factors that shape collaborative governance in an integrated system for collection of contributions for social health insurance, pension, and taxes from the informal sector. While is legislation crucial to support such collaboration, our research further underscores several other factors to consider for succefful governance of this intergrated system including strengthening leadership and coordination mechanisms, addressing communication gaps, and improving relational aspects, such as mitigating unbalanced power dynamics, self-interest, and building trust. Recognizing and actively addressing these factors can significantly contribute to the effective governance of the integrated system for contribution collection for social health insurance, pension, and taxes from the informal sector. Lastly, by fostering collaboration among the diverse institutions, we not only create a more inclusive contributory system but also contribute meaningfully to the achievement of universal health coverage and sustainable development targets.

## Supplementary Information

Supplementary Material 1.

## Data Availability

The author confirms that all data generated or analyzed during this study are included in this published article.

## Abbreviations

CGR: Collaborative governance regime
ILO: International Labor Organization
KII: Key informant interviews
LMICS: Low- and middle-income countries
MoH: Ministry of Health
NAPSA: National pensions scheme authority
SDG: Sustainable development goal
SHI: Social health insurance
TWG: Technical working group
UHC: Universal health coverage
ZRA: Zambia revenue authority
