# Supplementary material for: Collaborative governance of an integrated system for collecting contributions for social health insurance, pension, and taxes from the informal sector: a synthesis of stakeholder perspectives
Source: BMC Health Serv Res. 2024 Oct 17;24:1253. doi: 10.1186/s12913-024-11634-4 (PMC11487770; doi:10.1186/s12913-024-11634-4)
Supplement: Supplementary file 1 — Supplementary Material 1. [file 12913_2024_11634_MOESM1_ESM.docx]

**Appendix I – Interview guide for cooperating partners**

- - 1. Could you tell us more about your organization’s roles and its mandate?
    2. How do you work with the government in the informal sector/economy of Zambia?
    3. What are the challenges you face when working in the informal sector and how do you normally deal with them as an organization in the Zambia office?
    4. What do you think of Zambian organizations/ministries collaborating to create integrated systems?
    5. How do you think your organization would benefit from the idea of employing an integrated system?
    6. Are there some challenges that you would anticipate seeing? What would be some of the challenges of employing such a system?
    7. What are some of the interests that would inhibit the creation of an integrated system with other organizations?
    8. What do you think are some of the strategies or mechanisms that can be put in place to support the creation of an integrated system?
    9. How do you think this system could capture the informal sector to make them visible?
    10. What are some of the already existing platforms that could be utilized to support integration?

**Appendix II – Interview guide for government institutions**

- - 1. Could you tell us more about what your organization/Ministry does or is responsible for?
    2. How do you work in the informal sector and what policies guide you in this work?
    3. What are the challenges you face when dealing with the informal sector and how do you normally deal with them as an organization?
    4. What do you think of creating integrated systems by collaborating with other organizations to work effectively in the informal sector?
    5. How do you think your organization would benefit from the idea of employing an integrated system?
    6. Are there some challenges that you would anticipate? What would be some of the challenges of employing such a system?
    7. What are some of the interests that would block the creation of an integrated system with other organizations?
    8. What do you think are some of the strategies or mechanisms that can be put in place to support the creation of an integrated system?
    9. How do you think this system could capture the informal sector to make them visible?
    10. What are some of the already existing platforms that could be utilized to support integration?

**Appendix III – Interview guide for the informal sector**

1. Could you tell us more about what your organization does or is responsible for?
2. How do you work and what policies guide you in this work?
3. What are the challenges you face when dealing with when doing tour work and how do you normally deal with them as an organization?
4. What do you think about collaborating with other organizations to work effectively in the informal sector?
5. How do you think your organization would benefit from collaboration or the idea of employing an integrated system?
6. Are there some challenges that you would anticipate? What would be some of the challenges of employing such a system?
7. What are some of the interests that would inhibit the creation of an integrated system with other organizations?
8. What do you think are some of the strategies or mechanisms that can be put in place to support the creation of an integrated system?
9. How do you think this system could capture the informal sector to make them visible?
10. What are some of the already existing platforms that could be utilized to support integration?
